# The developing family doctor system: evidence from the progress of the family doctor signing service from a longitudinal survey (2013–2016) in Pudong New Area, Shanghai

**DOI:** 10.1186/s12875-020-01353-0

**Published:** 2021-01-08

**Authors:** Shanshan Liu, Yan Liu, Tao Zhang, Luan Wang, Jiaoling Huang, Hong Liang, Gang Chen, Chengjun Liu, Yimin Zhang

**Affiliations:** 1 Pudong Institute for Health Development, Shanghai, China; 2 School of Public Health, Fudan University, Shanghai, China; 3 Health Inspection Agency of Shanghai Pudong New Area Health Commission, Shanghai, China; 4 Jinyang Community Health Service Center of Pudong New Area, Shanghai, China; 5 Shanghai Jiao Tong University Affiliated Sixth People’s Hospital, Shanghai, China; 6 School of Medicine, Shanghai Jiao Tong University, Shanghai, China; 7 School of Social Development and Public Policy, Fudan University, Shanghai, China; 8 Eye and dental diseases prevention & treatment of Pudong new area, Shanghai, China

**Keywords:** Family doctor, Contract, Medical service, Health management, Community health service centers

## Abstract

**Background:**

The family doctor system is a vital part of China’s national medical and health system reform. Evidence of the degree of implementation of the family doctor system is required to assist managers and policy makers in Pudong with resource allocation planning. This study analyzed changes in indicators (family doctor team construction, contracted medical services, health management services and so on) over time to evaluate the progress of the family doctor system in Pudong.

**Methods:**

We used a cross-sectional design with an online questionnaire survey to collect 3-year (2013–2016) consecutive data. The online questionnaires were completed by the doctors responsible for information reporting in each community health service center of Pudong. The data were sorted, and the indices calculated and analyzed using descriptive statistics and statistical tests.

**Results:**

The proportion of registered general practitioners increased each year, from 50.8% in 2013 to 66.5% in 2016; this difference was statistically significant (*P* = 0.000). The number of family doctors per 10,000 permanent residents rose each year, from 1.7 in 2013 to 2.1 in 2016. The rate of contracted household residents was 55.7% in 2013 and increased to 71.7% in 2016, with the difference being significant in different years (*P* = 0.012). Analysis of referral services showed the people times of contracted residents transferring to higher-level hospitals from family doctors increased each year, from 172,734 in 2013 to 341,615 in 2016; differences among different regions were statistically significant for 2013–2016. The rate of health screening for contracted residents also increased each year, with statistically significant differences in different years (*P* = 0.000). The rate of health assessment interventions for contracted residents rose each year, with statistically significant differences in different years (*P* = 0.003).

**Conclusions:**

The family doctor signing service in Pudong made headway in general practitioner availability, contract service rate of household residents, and providing health management services. However, problems included family doctor shortages and limited supporting policies, especially in rural and suburban areas compared with urban divisions. Increasing the enrollment rate of family doctors and speeding up the implementation of “contract service fees” are key tasks for the sustainable development of the family doctor system in Pudong.

**Supplementary Information:**

The online version contains supplementary material available at 10.1186/s12875-020-01353-0.

## Background

Changes in medical modes and population aging resulted in a “blowout” of chronic diseases. The long-term survival of patients with chronic diseases requires good treatment, continuity of services, comprehensive community interventions, and personalized services. Furthermore, with the increasingly specialized and refined development of medical technology, a mismatch emerged between “high health demand” and “high service technology.” In reality, many patients experience difficulty finding corresponding medical service technology, meaning many cases require emergency treatment.

In China, promoting family doctor contracting services was a key breakthrough in implementing a graded diagnosis and treatment system. Through the family doctor contract service system, family doctors provide comprehensive services to help patients who lack professional knowledge to improve their ability to appropriately choose medical institutions and obtain long-term coordinated healthcare. To date, over 50 countries and regions have implemented a family doctor system, including the United Kingdom, Cuba, Australia, the United States, and Canada [1–6]. Although the healthcare systems, service modes, and operation mechanisms of family doctor systems differ in different countries, the family doctor system occupies an important position in medical and health service systems. There are also some common practices and characteristics across family doctor systems. For example, the Danish General Practitioners Association [7] began to sign healthcare contracts with residents in 1973 and in Holland, residents must choose a general practitioner for signing each year [8]. There is a clear indication of the number of residents that family doctors should sign with, which is set at about 2000 people.

Development of the family doctor system in Shanghai began in 2003, with pilot work for service mode conversion implemented in Jing’an District, Changning District, Huangpu District, Pudong New Area, and Zhabei District. The public has reached a certain understanding of family doctors, and various highlights and experiences have been reported [9–12]. Pudong implemented the family doctor system in 2010, and has focused on perfecting the management system, working mechanism innovations, optimizing service patterns and content, and promoting community health reform in general.

However, problems have been identified that require resolution, such as an inadequate contract service rate, family doctor shortages, and the absence of supporting policies [13–15]. This study aimed to investigate the degree of implementation of the family doctor system in Pudong based on several key indicators: general practitioner/family doctor team construction, medical service coverage and contracting, service provision status, and health management services. It is important to clarify barriers to strengthening the family doctor system to provide reference information for related health policy.

## Methods

### Study design

This study used a cross-sectional design, with data collected via an online questionnaire survey.

### Respondents

In total, 45 community health service centers (CHSCs) in Pudong New Area of China were included in our survey. Based on economic development level and geographical region, we classified CHSCs into urban divisions, suburban districts, and rural areas.

### Data collection

Quantitative data statistics were obtained from the online questionnaire used in the “Pudong New Area Family Doctors Annual Report” for CHSCs. Some data were obtained by searching the CNKI, SCIE, and VIPIN databases, Elsevier, and governmental statistics reports, setting “family doctor system” as the search term. These data included reviews, empirical studies, and proceedings, and were used in designing the questionnaire. At first, 60 indices were selected in the questionnaire, then according to expert consultation and pre-investigation experiment, 54 indices were formed. Finally, the questionnaire covered six parts: service coverage, staff, providing contract status, providing medical services, providing health management services, and providing other services with 54 indices (see Questionnaire in the additional file). The online questionnaires were completed by the doctors who were responsible for information reporting in each CHSC. Moreover, all data were reviewed by the director of each CHSC. The response rate of the survey was 100%, and investigation data concerning family doctor services for 45 CHSCs were included in this analysis. We analyzed consecutive data for 3 years (2013–2016). These data were sorted and the significance of each index was analyzed. The specific definitions for each index in this study were as follows.

- Proportion of registered general practitioners (%) = number of registered general practitioners / number of clinical (assistant) physicians.
- The proportion of nurses (%) = number of nurses included in the family doctor team / number of nurses in the community.
- Doctor to nurse ratio (%) = number of clinical (assistant) physicians / number of nurses in the community.
- Village physicians ratio (%) = number of village physicians included in the family doctor team / number of clinical (assistant) physicians.
- Family doctors per 10,000 permanent residents (N/10,000) = number of family doctors / number of permanent residents in the community / 10,000.
- Coverage of community health service stations (%) = number of community health service stations providing family doctor services / number of community health service stations.
- Coverage of the proportion of village clinics (%) = number of village clinics providing family doctor services / number of village clinics.
- Rate of contracted permanent residents (%) = number of permanent residents signing services / number of permanent residents.
- Rate of contracted household residents (%) = number of household residents signing services / number of household residents.
- Rate of contracted resident families (%) = number of permanent families signing services / number of permanent families.
- Proportion of contracted residents’ visits in total outpatient visits per year (%) = number of residents signing services who visited the community / number of residents signing services.
- Outpatient appointment rate per year (%) = number of appointments for outpatients (persons) / number of outpatients in the community health service center during the year.
- Rate of contracted residents establishing profile (%) = number of contracted residents’ electronic health records / number of contract residents.
- Ratio of health records dynamic updates (%) = number of contracted residents’ electronic health records updates / number of contracted residents’ electronic health records.
- Rate of contracted residents’ health screening (%) = number of contracted residents who conducted health screening / number of contract residents.
- Rate of contracted residents’ health assessment interventions (%) = number of contract residents who received targeted intervention guidance programs / number of contracted residents who received health status and health needs assessment services.

### Statistical analyses

Data were analyzed using SPSS version 18.0. Descriptive statistics were calculated for CHSC characteristics, and a scoring ratio (%) was used to explain the situation of the family doctor system implementation. Chi-square tests were used to examine comparisons of the variables between groups, and the threshold of statistical significance (α) was set at *P* < 0.05 (two-tailed). Pairwise comparisons of multiple sample rates were performed: α’ = α/[k*(k-1)/2 + 1]. The Cochran-Mantel-Haenszel test was used to assess the impact of time trends.

## Results

### Characteristics of CHSCs

There are 45 CHSCs in Pudong New Area, including 13 urban divisions, 8 suburban districts, and 24 rural areas. In 2013 and 2014, 42 centers met the standards for community standardization, but all 45 centers had reached this standard since 2015. The number of certified general practitioner training bases increased from 4 in 2013 to 8 in 2016. Since 2014, all CHSCs were designated as medical insurance agencies. Some centers had achieved zero difference sales of essential drugs, which increased to 13 centers in 2016 (see Table 1).

**Table 1:** Sociological characteristic of CHSCs in Pudong from 2013 to 2016

Characteristics	Year			
	2013	2014	2015	2016
Regional classification (N/%)				
Urban division	13(28.9)	13(28.9)	13(28.9)	13(28.9)
Suburban district	8(17.8)	8(17.8)	8(17.8)	8(17.8)
Rural area	24(53.3)	24(53.3)	24(53.3)	24(53.3)
Standardized construction (N/%)				
Yes	42(93.3)	42(93.3)	45(100.0)	45(100.0)
No	3(6.7)	3(6.7)	0	0
GP practice training base (N/%)				
Yes	4(8.9)	6(13.3)	7(15.6)	8(17.8)
No	41(91.1)	39(86.7)	38(84.4)	37(82.2)
Medical insurance fixed-points(N/%)				
Yes	44(97.8)	45(100.0)	45(100.0)	45(100.0)
No	1(2.2)	0(0.0)	0(0.0)	0(0.0)
Zero difference sales of essential drugs (N/%)				
Yes	0(0.0)	6(13.3)	10(22.2)	13(28.9)
No	45(100.0)	39(86.7)	35(77.8)	32(71.1)
Number of employees on the job (N)	6381	6562	6707	6793
Real number of beds (N)	3362	3220	3207	3188
Housing construction area (m^2^)	345,045	352,519	348,223	344,876

### Family doctor team construction

The proportion of registered general practitioners in family doctor teams increased each year, from 50.8% in 2013 to 66.5% in 2016, with the differences being statistically significant (*P* = 0.000). The proportion of nurses in family doctor teams also increased each year, from 40.2% in 2013 to 46.1% in 2016; however, this difference was not significant (*P* = 0.861), although different regions showed differences for 2016 (*P* = 0.049). The doctor to nurse ratio changed from 1.4 in 2013 to 1.2 in 2016, but no statistically significant difference was found in different years (*P* = 0.120). The ratio of village physicians in the team decreased each year, whereas the number of family doctors per 10,000 permanent residents increased each year (from 1.7 in 2013 to 2.1 in 2016) (see Table 2).

**Table 2:** Family doctor staff and team construction

Year	Regional classification	Family doctor team construction					
		Proportion of registered general practitioners (%)	The proportion of nurses (%)	Doctor to nurse ratio (%)	Village physicians ratio (%)	Number of family doctors per ten thousand permanent residents (N/10000)	Number of family doctor service teams (N)
2013	Urban division	375/816(46.0)	187/550(34.0)	816/550(1.5)	0(0.0)	318(2.0)	82
	Suburban district	231/417(55.4)	112/315(35.6)	417/315(1.3)	75/114(65.8)	162(1.0)	42
	Rural area	523/989(52.9)	345/737(46.8)	989/737(1.3)	638/720(88.6)	419(1.9)	172
	Total	1129/2222(50.8)	644/1602(40.2)	2222/1602(1.4)	713/834(85.5)	899(1.7)	296
	Statistics (P) ^a^	1.174(0.319)	1.939(0.157)	0.430(0.653)	–	–	0.619(0.544)
2014	Urban division	404/820(49.3)	213/562(37.9)	820/562(1.5)	0(0.0)	337(2.1)	84
	Suburban district	246/422(58.3)	114/324(35.2)	422/324(1.3)	74/112(66.1)	164(1.1)	43
	Rural area	589/1000(58.9)	384/742(51.8)	1000/742(1.4)	639/719(88.9)	488(2.1)	179
	Total	1239/2242(55.3)	711/1628(43.7)	2242/1628(1.4)	713/831(85.8)	989(1.8)	306
	Statistics (P) ^a^	1.743(0.187)	2.189(0.125)	0.450(0.640)	–	–	0.672(0.516)
2015	Urban division	431/787(54.8)	248/579(42.8)	787/579(1.4)	0(0.0)	359(2.3)	94
	Suburban district	286/450(63.6)	114/382(29.8)	450/382(1.2)	74/123(60.2)	190(1.3)	42
	Rural area	639/999(64.0)	413/805(51.3)	999/805(1.2)	632/710(89.0)	522(2.2)	167
	Total	1356/2236(60.6)	775/1766(43.9)	2236/1766(1.3)	706/833(84.8)	1071(2.0)	303
	Statistics (P) ^a^	1.656(0.203)	2.846(0.069)	0.521(0.598)	–	–	0.375(0.689)
2016	Urban division	501/794(63.1)	283/586(48.3)	794/586(1.4)	0(0.0)	406(2.6)	101
	Suburban district	310/445(69.7)	116/412(28.2)	445/412(1.1)	63/114(55.3)	200(1.4)	42
	Rural area	698/1029(67.8)	450/843(53.4)	1029/843(1.2)	623/703(88.6)	551(2.2)	176
	Total	1509/2268(66.5)	849/1841(46.1)	2268/1841(1.2)	686/817(84.0)	1157(2.1)	319
	Statistics (P)^a^	0.701(0.502)	3.233(0.049)^c^	1.948(0.155)	–	–	0.426(0.656)
Statistics (P) ^b^		7.731(0.000)^d^	0.250(0.861)	1.974(0.120)	0.001(1.000)	2.503(0.061)	0.077(0.972)

^a^Refers to the comparison among regions in the same year

^b^Refers to the comparison among different years

^c^Suburban district VS Rural area (*P* = 0.015)

^d^2013 VS 2015 (*P* = 0.005), 2013 VS 2016 (*P* = 0.000), 2014 VS 2016 (*P* = 0.001)

### Family doctor system coverage and contract

The proportion of CHSCs covered by the family doctor system increased each year, from 97.2% in 2013 to 100.0% in 2016. Since 2014, village clinics achieved full coverage of the family doctor system. The contract rate of permanent residents increased each year (39.1% in 2013 and 45.9% in 2016), but the difference was not significant in different years (*P* = 0.130). The contract rate for household residents was 55.7% in 2013 and rose to 71.7% in 2016, with the difference being statistically significant in different years (*P* = 0.012); there were also statistically significant differences among different regions in 2013, 2014, and 2015 (*P* = 0.002, *P* = 0.006, and *P* = 0.001, respectively). The contract rate of resident families also increased each year; the differences were significant among different regions, but were not significant in different years (see Table 3).

**Table 3:** Family doctor system coverage and contract situation

Year	Regional classification	Family doctor system coverage and contract situation				
		Coverage of community health service stations (%)	Covering the proportion of village clinics (%)	Rate of contract permanent residents (%)	Rate of contract household residents (%)	Rate of contract resident families(%)
**2013**	Urban division	52/53(98.1)	0(0.0)	7,123,145/1659332(42.9)	501,629/1135807(44.2)	418,829/582929(71.9)
	Suburban district	29/29(100.0)	34/42(81.0)	449,309/1177420(38.2)	324,248/448081(72.4)	206,551/433250(47.7)
	Rural area	23/25(92.0)	269/275(97.8)	834,083/2262285(36.9)	655,649/1075640(61.0)	380,480/797488(47.7)
	Total	104/107(97.2)	303/317(95.6)	1,995,706/5099037(39.1)	1,481,526/2659528(55.7)	1,005,860/1813667(55.5)
	Statistics (P) ^a^	3.477(0.176)	–	0.102(0.903)	7.333(0.002) ^c^	4.327(0.020)^f^
**2014**	Urban division	52/53(98.1)	0(0.0)	763,558/1646658(46.4)	544,900/1014883(53.7)	455,518/585660(77.8)
	Suburban district	30/30(100.0)	40/40(100.0)	482,950/1189133(40.6)	339,901/432888(78.5)	226,946/438707(51.7)
	Rural area	24/25(96.0)	272/272(100.0)	927,679/2268178(40.9)	680,973/950174(71.7)	421,602/809522(52.1)
	Total	106/108(98.2)	312/312(100.0)	2,174,187/5103969(42.6)	1,565,774/2397945(65.3)	1,104,066/1833889(60.2)
	Statistics (P) ^a^	1.201(0.549)	–	0.144(0.866)	5.816(0.006) ^d^	5.278(0.009) ^g^
**2015**	Urban division	52/52(100.0)	0(0.0)	784,209/1656623(47.3)	571,326/1044387(54.7)	600,359/469673(78.2)
	Suburban district	31/31(100.0)	38/38(100.0)	492,349/1247302(39.5)	332,156/448075(74.1)	460,969/233035(50.6)
	Rural area	27/27(100.0)	270/270(100.0)	953,766/2292078(41.6)	704,119/944568(74.5)	443,276/823395(53.8)
	Total	110/110(100.0)	308/308(100.0)	2,230,324/5196003(42.9)	1,607,601/2437030(66.0)	1,145,984/1884723(60.8)
	Statistics (P) ^a^	–	–	0.247(0.782)	8.170(0.001) ^e^	5.870(0.006) ^h^
**2016**	Urban division	53/53(100.0)	0(0.0)	881,547/1608690(54.8)	688,211/1035593(66.5)	569,449/592532(96.1)
	Suburban district	30/30(100.0)	32/32(100.0)	510,645/1196288(42.7)	347,923/447566(77.7)	248,696/448164(55.5)
	Rural area	27/27(100.0)	268/268(100.0)	973,462/2349714(41.4)	711,122/955129(74.5)	457,366/840055(54.4)
	Total	110/110(100.0)	300/300(100.0)	2,365,654/5154692(45.9)	1,747,256/2438288(71.7)	1,275,511/1880751(67.8)
	Statistics (P) ^a^	–	–	1.572(0.220)	1.245(0.298)	7.605(0.002) ^i^
Statistics (P)^b^		5.604(0.133)	0.253(0.859)	1.910(0.130)	3.783(0.012)^j^	1.696(0.170)

^a^Refers to the comparison among regions in the same year

^b^Refers to the comparison among different years

^c^Urban division VS Suburban district (*P* = 0.001), Urban division VS Rural area (*P* = 0.005).

^d^Urban division VS Suburban district (*P* = 0.004), Urban division VS Rural area (*P* = 0.008).

^e^Urban division VS Suburban district (*P* = 0.005), Urban division VS Rural area (*P* = 0.000)

^f^Urban division VS Suburban district (*P* = 0.006), Urban division VS Rural area (*P* = 0.005).

^g^Urban division VS Suburban district (*P* = 0.020), Urban division VS Rural area (*P* = 0.004).

^h^Urban division VS Suburban district (*P* = 0.004), Urban division VS Rural area (*P* = 0.006).

^i^Urban division VS Suburban district (*P* = 0.003), Urban division VS Rural area (*P* = 0.001)

^j^2013 VS 2014 (*P* = 0.030), 2013 VS 2015 (*P* = 0.024), 2013 VS 2016 (*P* = 0.001)

### Family doctors offering contracted medical services

The proportion of contracted resident visits in all outpatient visits was 37.4% in 2013 and 40.5% in 2016, but the difference was not statistically significant. The outpatient appointment rate per year was low, with that in rural areas being higher compared with urban divisions and suburban districts, although the difference was not statistically significant each year. The number of contracted resident visits to family doctors per year showed a downward trend, with the trend being more notable in the urban division compared with suburban districts and rural areas; the differences among regions were statistically significant in 2013, 2014, and 2015. The people times of referrals to higher level hospitals through family doctors per year increased each year. However, there were more referrals in rural areas than in suburban districts and urban divisions, and these differences were statistically significant in different years. Referrals from higher level hospitals through family doctors per year in suburban districts were higher compared with the urban divisions and rural areas in 2013 and 2014, with these differences being statistically significant (*P* = 0.000 and *P* = 0.001, respectively) (see Table 4).

**Table 4:** Family doctor providing medical service among contract residents

Year	Regional classification	Family doctor providing orderly medical service				
		Proportion of contract residents visits in the total outpatient visits per year(%)	Outpatient appointment rate per year(%)	Number of contract residents visits each family doctor per year (N)	People times of referral to higher level hospital by family doctor per year (N)	People times of referral from higher level hospital by family doctor per year (N)
**2013**	Urban division	48.0	0.8	9996	18,799	1265
	Suburban district	31.9	2.1	6501	39,520	6232
	Rural area	29.0	2.7	4277	114,415	1643
	Total	37.4	1.8	6701	172,734	9140
	Statistics (P) ^a^	2.471(0.097)	1.800(0.178)	8.363(0.001) ^c^	2.644(0.038)^d^	11.479(0.000)^h^
**2014**	Urban division	44.3	1.4	8834	23,290	1579
	Suburban district	24.3	2.2	5228	48,602	6707
	Rural area	30.0	3.0	4238	188,509	2654
	Total	34.4	2.2	5968	260,401	10,940
	Statistics (P) ^a^	1.324(0.277)	1.377(0.264)	4.701(0.014)^j^	3.273(0.048) ^e^	7.725(0.001) ^i^
**2015**	Urban division	46.8	1.2	9310	26,013	3145
	Suburban district	23.3	1.9	4455	45,012	6516
	Rural area	37.5	4.9	5310	210,917	2977
	Total	38.3	2.8	6499	281,942	12,638
	Statistics (P) ^a^	1.827(0.173)	2.134(0.131)	5.551(0.007) ^k^	3.632(0.035)^f^	0.279(0.758)
**2016**	Urban division	50.5	1.7	8693	31,644	5201
	Suburban district	26.3	1.4	4957	65,367	8597
	Rural area	38.5	3.7	5617	244,604	4217
	Total	40.5	2.5	6582	341,615	18,015
	Statistics (P) ^a^	2.115(0.133)	2.055(0.141)	2.286(0.114)	4.330(0.020)^g^	0.369(0.693)
Statistics(P)^b^		1.116(0.344)	1.074(0.361)	0.143(0.934)	2.154(0.095)	1.030(0.380)

^a^Refers to the comparison among regions in the same year

^b^Refers to the comparison among different years

^c^Urban division VS Rural area (*P* = 0.000), Urban division VS Suburban district (*P* = 0.020), Suburban district VS Rural area (*P* = 0.015).

^d^Urban division VS Rural area (*P* = 0.002), Suburban district VS Rural area (*P* = 0.000).

^e^Urban division VS Rural area (*P* = 0.010), Suburban district VS Rural area (*P* = 0.000).

^f^Urban division VS Rural area (*P* = 0.005), Suburban district VS Rural area (*P* = 0.000).

^g^Urban division VS Rural area (*P* = 0.001), Suburban district VS Rural area (*P* = 0.000)

^h^Urban division VS Suburban district (*P* = 0.000), Suburban district VS Rural area (*P* = 0.000).

^i^Urban division VS Suburban district (*P* = 0.000), Suburban district VS Rural area (*P* = 0.000)

^j^Urban division VS Suburban district (*P* = 0.014), Urban division VS Rural area (*P* = 0.005).

^k^Urban division VS Suburban district (*P* = 0.003), Urban division VS Rural area (*P* = 0.013)

### Family doctors offering health management services

The rate of contracted residents establishing a profile was 55.7% in 2013 and increased to 96.8% in 2016. The ratio of health record dynamic updates decreased each year, but the difference was not statistically significant. The rate of health screening for contracted residents increased each year, and the differences were statistically significant in different years (*P* = 0.000). Finally, the rate of health assessment interventions for contracted residents increased each year, and these differences were statistically significant in different years (*P* = 0.003) (see Table 5).

**Table 5:** Family doctor health management service

Year	Regional classification	Family doctor health management service			
		Rate of contract residents establishing profile (%)	Ratio of health records dynamic update (%)	Rate of contract residents health screening (%)	Rate of contract residents health assessment intervention (%)
**2013**	Urban division	69.1	64.9	46.9	92.4
	Suburban district	48.2	47.7	36.4	82.8
	Rural area	51.4	41.4	62.4	63.4
	Total	55.7	51.2	50.0	76.8
	Statistics (P) ^a^	0.666(0.519)	0.097(0.908)	1.685(0.198)	1.604(0.213)
**2014**	Urban division	55.2	39.4	60.5	93.5
	Suburban district	49.4	35.6	45.8	85.6
	Rural area	47.0	35.9	68.0	68.4
	Total	50.2	37.1	59.7	80.7
	Statistics (P) ^a^	0.246(0.783)	0.006(0.994)	1.339(0.273)	1.517(0.231)
**2015**	Urban division	51.2	29.2	75.7	96.3
	Suburban district	46.8	29.1	58.4	91.7
	Rural area	48.2	38.9	74.4	75.9
	Total	48.8	33.1	70.7	86.8
	Statistics (P) ^a^	0.262(0.771)	0.885(0.420)	0.715(0.495)	0.665(0.519)
**2016**	Urban division	95.4	41.6	77.7	100.0
	Suburban district	99.1	31.0	60.8	94.2
	Rural area	97.0	36.5	77.7	74.9
	Total	96.8	37.2	72.1	91.8
	Statistics (P) ^a^	0.002(0.998)	0.900(0.414)	0.392(0.678)	0.435(0.650)
Statistics(P)^b^		1.404(0.243)	2.168(0.093)	8.570(0.000)^c^	4.876(0.003)^d^

^a^Refers to the comparison among regions in the same year

^b^Refers to the comparison among different years

^c^2013 VS 2015 (*P* = 0.003), 2013 VS 2016 (*P* = 0.000), 2014 VS 2016 (*P* = 0.001).

^d^2013 VS 2015 (*P* = 0.019), 2013 VS 2016 (*P* = 0.000), 2014 VS 2016 (*P* = 0.013)

## Discussion

In China, the basic policy for the family doctor system is to be the community’s first diagnosis system. International and domestic practice showed that promoting family doctor signing services in primary medical and healthcare institutions offered a feasible way to protect and maintain public health [16–20]. This method is conducive to changing the model of medical and health services and allows family doctors to act as health gatekeepers. Since the State Council issued the Guiding Opinions on Establishing a General Practitioner System in 2011, pilots of different forms of family doctor signing services have been launched in various places, including team building, incentives, and evaluations [21–26]. As early as November 2010, Pudong established the family doctor system, and since April 2012 all CHSCs in Pudong have comprehensively implemented the system. However, some issues emerged with the system implementation, such as who should provide family doctor signing services. The professionals engaged in family doctor services differ slightly across various countries, but the qualification is strictly regulated. For example, in the United Kingdom, family doctors need to complete medical professional training (including in all departments of hospital rotation training), and physicians registered with the Royal Society of Medicine are comprehensive primary healthcare personnel with clinical skills [27, 28]. In the United States, general practitioners must first demonstrate good learning achievement and medical college clinical assessment, participate in the national unified standard exam, and then complete an interview that covers issues such as personal vision to determine whether their life and value outlooks are suited to general practice [29, 30].

This study showed that family doctors in Pudong were the persons with first responsibility for providing contracted health services to the public. Family doctors were mainly registered general practitioners (including assistant general practitioners and traditional Chinese medicine general practitioners), as well as some village doctors who were deemed capable. With the development of family doctor teams, contracted service teams based around general practitioners were gradually formed, which mainly included family doctors, community nurses, public health physicians (assistant public health physicians), and village doctors. In this study, the proportion of registered general practitioners in these teams was 50.8% in 2013, but rose to 66.5% in 2016. Furthermore, with the “National Comprehensive Reform Pilot Area for the Development of Traditional Chinese Medicine,” Pudong introduced traditional Chinese medicine physicians into family doctor teams to provide special traditional Chinese medicine services. Our study found there were 236 traditional Chinese medicine physicians working as family doctors, which accounted for 19.4% of family doctor teams by the end of 2017. In addition, non-medical community assistants and community volunteers were also recruited to join the teams. In these teams, family doctors are responsible for task distribution and management of team members, and work closely with other specialists and medical technicians to provide high-quality services to contracted residents.

The present study showed that the proportion of registered general practitioners in the family doctor teams increased each year, from 50.8% in 2013 to 66.5% in 2016. Pudong had 2.1 general practitioners per 10,000 permanent residents in 2016; however, the target of the “13th Five-Year Plan for the Reform and Development of Health and Family Planning in Shanghai” is set at 4–5 general practitioners per 10,000 permanent residents. Given the current population of 5.50 million permanent residents in Pudong, at least 1045 more general practitioners are needed to achieve full population coverage. In 2014, Pudong issued “interim measures on further strengthening the construction of rural health talent team” (referred to as “eight health polices”) for rural and suburban personnel in CHSCs [31]. General practitioners in CHSCs in rural areas and suburban districts received 2000–6000 RMB/month as a work allowance, which increased work enthusiasm. However, the effect of the incentive policy may take some time to appear, and as the permanent population of Pudong is increasing each year, family doctor signing services continue to face the problem of a shortage of general practitioners.

Family doctors are the main body providing contracted services, and this study raised questions as to what form services would take and how residents sign contracts with family doctors in Pudong. Our investigation showed that residents or families can voluntarily choose one family doctor team with which to sign a service agreement. The service period for each contract is 1 year in principle. In this study, the rate of contracted permanent residents was 39.1% in 2013 and rose to 45.9% in 2016 (no statistically significant difference), whereas the rate of contracted household residents increased each year, reaching 71.7% in 2016 (significant difference from 2013 to 2016). At the same time, we found the rate of contracted household residents in suburban districts was higher than that in urban divisions and rural areas. However, the rate of contracted household residents rapidly increased. A potential reason for this observation is the relative shortage of medical resources in rural and suburban areas, which made it easier to implement the family doctor system. In addition, the stimulus policy for general practitioners (“eight health polices”) in the rural areas of Pudong took effect. Moreover, Since 2014, the “1 + 1 + 1” medical institution combination contract (a community health service center, a district-level hospital, and a municipal-level hospital) has been explored in Shanghai; therefore, when signing with a family doctor team in the community, people or families can also be recommended to a secondary or tertiary hospital for treatment in the combination medical institutions [32, 33]. This means family doctors not only provide basic health services, but also provide other services such as appointment services, two-way referral services, health counseling and education, and door-to-door services. This means family doctors have a heavy workload, which makes it difficult to guarantee service quality in Pudong. The National Health Commission and the State Administration of Traditional Chinese Medicine jointly issued “Guiding Opinions on Regulating the Management of Family Doctor Contracting Services” in 2018. This document proposed that the number of residents signed to each family doctor should not exceed 2000 in principle. However, we found significant regional differences in the number of two-way referrals by family doctors per year in Pudong. There were more referrals in rural areas than in suburban districts and urban divisions, which reflected the large demand for medical services in remote areas. Therefore, it is necessary to adopt a regionally differentiated family doctor system promotion model.

### Strengths and limitations

The study includes all community health service centers in Pudong New Area, and the sample is representative. The data used in this study were collected directly through online reporting, which avoided some information bias caused by manual completion and on-site answers, but all data were completed and reported by CHSC information staff, so the selective information bias might have occurred. Thence in the next study, to get more objective and accurate data, the relevant information for family doctors in each CHSC can be directly retrieved through the medical data information platform after obtaining the information collection authority.

## Conclusions

The family doctor signing service in Pudong made headway in registered general practitioner availability, the contract service rate of household residents, and providing health management services. However, we identified problems in terms of a shortage of family doctors and limited supporting policies, especially in rural and suburban areas compared with urban divisions. Therefore, to ensure the sustainable development of the family doctor system, a series of “get in, stay, and do well” tasks need to be continued. The enrollment of family doctors must be increased, and strategies such as providing apartments, children’s education, and one-time incentives to attract and retain family doctors may be needed. Speeding up the implementation of “contract service fees” may also improve the enthusiasm of family doctors to extend signing services and reflect rewarding work and rewards.

## Supplementary Information

**Additional file 1.** Questionnaire of Pudong New Area Family Doctors Annual Report, which was used to investigate the situation of family doctors in community health service centers from year 2013 to 2016.

## Data Availability

The datasets used and/or analyzed during this study are available from the corresponding author on reasonable request.

## Abbreviation

CHSCs: Community health service centers.
