# Supplementary material for: The developing family doctor system: evidence from the progress of the family doctor signing service from a longitudinal survey (2013–2016) in Pudong New Area, Shanghai
Source: BMC Fam Pract. 2021 Jan 8;22:11. doi: 10.1186/s12875-020-01353-0 (PMC7792058; doi:10.1186/s12875-020-01353-0)
Supplement: Supplementary file 1 — Additional file 1. Questionnaire of Pudong New Area Family Doctors Annual Report, which was used to investigate the situation of family doctors in community health service centers from year 2013 to 2016. [file 12875_2020_1353_MOESM1_ESM.doc]

**Pudong New Area Family Doctors Annual Report**

Name of community health service center:

|  | Project | 2013 | 2014 | 2015 | 2016 |
| --- | --- | --- | --- | --- | --- |
| **1** | **Coverage** |  |  |  |  |
| 1.1 | Number of community health service stations |  |  |  |  |
| 1.1.1 | Among them: the family doctor covering the number of community health service stations |  |  |  |  |
| 1.2 | Number of village clinics |  |  |  |  |
| 1.2.1 | Among them: the family doctor covering the village health room number |  |  |  |  |
| **2** | **The staff** |  |  |  |  |
| 2.1 | Number of clinicians (person) |  |  |  |  |
| 2.1.1 | Among them: registered general practitioner (person) |  |  |  |  |
| 2.1.2 | Number of family doctors (person) |  |  |  |  |
| 2.2 | Number of Chinese doctors (person) |  |  |  |  |
| 2.2.1 | Among them: the number of traditional Chinese medicine practitioners (people) |  |  |  |  |
| 2.2.2 | Number of family doctors (person) |  |  |  |  |
| 2.3 | Number of community nurses (person) |  |  |  |  |
| 2.3.1 | Among them: the family doctor team of community nurses (people) |  |  |  |  |
| 2.4 | Under the jurisdiction of rural doctors (person) |  |  |  |  |
| 2.4.1 | The number of rural doctors (people) in the family doctor team |  |  |  |  |
| 2.5 | Number of family doctor team (unit) |  |  |  |  |
| 2.5.1 | Among them: the number of non medical community assistants included in the team (people) |  |  |  |  |
| 2.5.2 | Number of volunteers into the team (person) |  |  |  |  |
| 2.5.3 | Number of social workers into the team (person) |  |  |  |  |
| 3 | **Signing status** |  |  |  |  |
| 3.1 | Number of resident households in the community (home) |  |  |  |  |
| 3.1.1 | Among them: the number of signed home (home) |  |  |  |  |
| 3.2 | Number of permanent residents in the community (person) |  |  |  |  |
| 3.2.1 | Among them: the number of contracts (person) |  |  |  |  |
| 3.3 | Number of residents in the community (people) |  |  |  |  |
| 3.3.1 | Among them: the number of contracts (person) |  |  |  |  |
| 3.4 | Number of registered residents living in the community (person) |  |  |  |  |
| 3.4.1 | Among them: the number of contracts (person) |  |  |  |  |
| 4 | **Providing orderly medical service** |  |  |  |  |
| 4.1 | The family doctor year cumulative admissions signed number of residents (person) |  |  |  |  |
| 4.2 | The family doctor year cumulative number of admissions for contracted residents (people) |  |  |  |  |
| 4.3 | Cumulative number of outpatient visits in the community health service center (person time) |  |  |  |  |
| 4.3.1 | Among them: the number of outpatient visits (person time) |  |  |  |  |
| 4.4 | Residents contracted by family doctors transferred to the hospital for a higher level of hospital years (people) |  |  |  |  |
| 4.4.1 | Among them: the number of people (through the appointment of referral referral platform) |  |  |  |  |
| 4.4.1.1 | Internal: transferred to the general specialist clinic |  |  |  |  |
| 4.4.1.2 | Transferred to the specialist clinic |  |  |  |  |
| 4.4.1.3 | Transferred to the ward |  |  |  |  |
| 4.4.2 | Among them: through the regional referral referral channels (TRIPS) |  |  |  |  |
| 4.4.2.1 | Internal: transferred to the general specialist clinic |  |  |  |  |
| 4.4.2.2 | Transferred to the specialist clinic |  |  |  |  |
| 4.4.2.3 | Transferred to the ward |  |  |  |  |
| 4.5 | Family doctor receiving from the hospital back to the hospital for a number of times the cumulative number of residents (people) |  |  |  |  |
| 4.5.1 | Among them: outpatient follow-up |  |  |  |  |
| 4.5.2 | Transfer to community hospital bed |  |  |  |  |
| 4.5.3 | Turn to the family bed |  |  |  |  |
| 4.5.4 | Home to home service |  |  |  |  |
| 5 | **Provide health management services** |  |  |  |  |
| 5.1 | The contracted residents standardized electronic health records established number (copies) |  |  |  |  |
| 5.1.1 | Among them: the number of records (copies of the cumulative dynamic update） |  |  |  |  |
| 5.2 | The number of family doctors and health advisory team accumulated answering telephone and other forms of the (people) |  |  |  |  |
| 5.3 | Number of residents who had received home health status and health needs assessment (person) |  |  |  |  |
| 5.3.1 | Of which: according to the results of the assessment to develop targeted intervention guidelines number (person) |  |  |  |  |
| 6 | **others** |  |  |  |  |
| 6.1 | Clinicians involved in the work of the family doctor |  |  |  |  |
| 6.2 | Electronic health record (copy) |  |  |  |  |
| 6.3 | Home service (person time) |  |  |  |  |
| 6.4 | Active telephone access (person time) |  |  |  |  |
| 6.5 | Media reports (Times) |  |  |  |  |
